# *N*-(2,6-Diisopropyl­phen­yl)formamide toluene 0.33-solvate

**DOI:** 10.1107/S1600536812017527

**Published:** 2012-04-28

**Authors:** Matthias Berger, Jan W. Bats, Norbert Auner

**Affiliations:** a Institut für Anorganische Chemie der Universität Frankfurt, Max-von-Laue-Strasse 7, D-60438 Frankfurt am Main, Germany; b Institut für Organische Chemie, Universität Frankfurt, Max-von-Laue-Strasse 7, D-60438 Frankfurt am Main, Germany

## Abstract

The crystal packing of the title compound, C_13_H_19_NO·0.33C_7_H_8_, shows a channel at [001], which contains grossly disordered toluene solvent mol­ecules. The angle between the benzene ring and the mean plane of the formamide group is 71.1 (1)°. The amide groups of neighbouring mol­ecules are connected by N—H⋯O hydrogen bonds, forming 2_1_ helical chains propagating along [001]. Mol­ecules are also connected by weak inter­molecular C—H⋯O hydrogen bonds, forming 6_1_ helices.

## Related literature

For the synthesis of the starting material, see: Krishnamurthy (1982 ▶); Hinter­mann (2007 ▶). For the crystal structures of related compounds, see: Stibrany & Potenza (2006 ▶); Chitanda *et al.* (2008 ▶); Omondi *et al.* (2008 ▶); Gowda *et al.* (2009 ▶). For the treatment of the disordered solvent, see: Spek (2009 ▶).
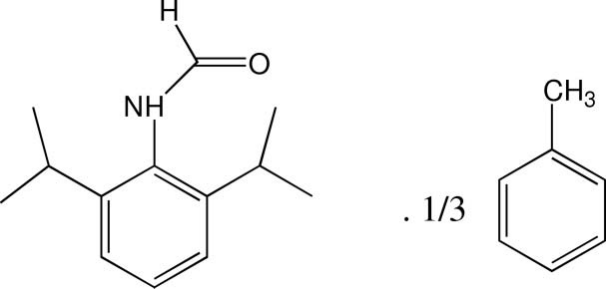


## Experimental

### 

#### Crystal data

C_13_H_19_NO·0.33C_7_H_8_
*M* *_r_* = 236.00
Hexagonal, 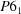

*a* = 16.9133 (6) Å
*c* = 8.4451 (4) Å
*V* = 2092.2 (2) Å^3^
*Z* = 6
Mo *K*α radiation
μ = 0.07 mm^−1^
*T* = 185 K
0.65 × 0.20 × 0.19 mm

#### Data collection

Siemens SMART 1K CCD diffractometer
Absorption correction: multi-scan (*SADABS*; Sheldrick, 2000 ▶) *T*_min_ = 0.933, *T*_max_ = 0.987
23771 measured reflections
1748 independent reflections
1503 reflections with *I* > 2σ(*I*)
*R*_int_ = 0.057

#### Refinement

*R*[*F* ^2^ > 2σ(*F* ^2^)] = 0.058
*wR*(*F* ^2^) = 0.125
*S* = 1.08
1748 reflections
144 parameters
1 restraint
H atoms treated by a mixture of independent and constrained refinement
Δρ_max_ = 0.16 e Å^−3^
Δρ_min_ = −0.16 e Å^−3^

### 

Data collection: *SMART* (Siemens, 1995 ▶); cell refinement: *SAINT* (Siemens, 1995 ▶); data reduction: *SAINT*; program(s) used to solve structure: *SHELXS97* (Sheldrick, 2008 ▶); program(s) used to refine structure: *SHELXL97* (Sheldrick, 2008 ▶); molecular graphics: *SHELXTL* (Sheldrick, 2008 ▶); software used to prepare material for publication: *SHELXL97*.

## Supplementary Material

Crystal structure: contains datablock(s) global, I. DOI: 10.1107/S1600536812017527/su2409sup1.cif

Structure factors: contains datablock(s) I. DOI: 10.1107/S1600536812017527/su2409Isup2.hkl

Supplementary material file. DOI: 10.1107/S1600536812017527/su2409Isup3.cml

Additional supplementary materials:  crystallographic information; 3D view; checkCIF report

## Figures and Tables

**Table 1:** Hydrogen-bond geometry (Å, °)

*D*—H⋯*A*	*D*—H	H⋯*A*	*D*⋯*A*	*D*—H⋯*A*
N1—H1*A*⋯O1^i^	0.80 (4)	2.05 (4)	2.826 (3)	164 (3)
C4—H4*A*⋯O1^ii^	0.95	2.56	3.418 (3)	150
C13—H13*A*⋯C3^i^	0.95	3.01	3.917 (4)	161
C13—H13*A*⋯C4^i^	0.95	3.03	3.973 (4)	173

Symmetry codes: (i) 
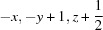
; (ii) 
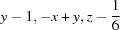
.

**Fig. 1.**
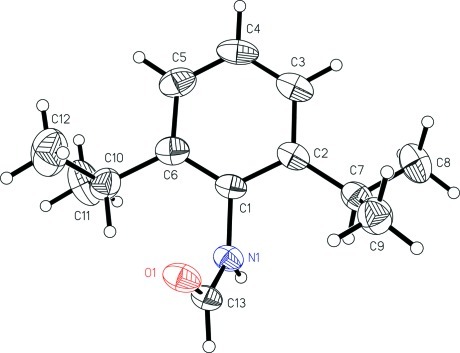
The molecular structure of the title molecule, showing the atom-labelling. Displacement ellipsoids are drawn at the 50% probability level.

**Fig. 2.**
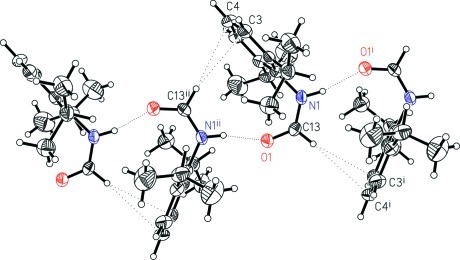
A view of the hydrogen bonded helical 21 chain of title molecules lying parallel to the c axis. Displacement ellipsoids are drawn at the 50% probability level. The hydrogen bonds are shown as dotted lines [symmetry codes: (i) -x, -y + 1, z + 0.5; (ii) -x, -y + 1, z - 0.5].

**Fig. 3.**
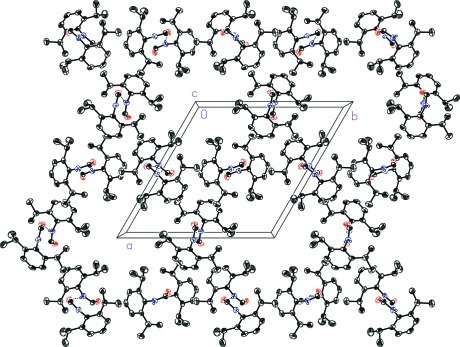
A view along the c axis of the crystal packing of the title compound, showing the solvent accessible channel along [0 0 1]. H atoms on C atoms have been omitted for clarity. Displacement ellipsoids are drawn at the 50% probability level. Intermolecular N—H···O hydrogen bonds are shown as dotted lines.

## supplementary crystallographic information

### Comment

The title compound was obtained as a byproduct from the synthesis of the *N*-heterocyclic carbene precursor 1,3-bis-(2,6-diisopropylphenyl)imidazolium chloride (Hintermann, 2007). Crystallization from a solution in toluene provided single crystals of the title compound, whose crystal structure is reported herein.

The molecular structure of the title molecule is shown in Fig. 1. The angle between the benzene ring and the mean plane of the formamide group is 71.1 (1)°. It is slightly smaller than the value of 77.4 (1)° reported for the crystal structure of the solvent-free compound (Stibrany & Potenza, 2006; Chitanda *et al.*, 2008). This non-planar geometry is required by steric repulsions between the formamide group and the isopropyl substituents. An almost planar molecule has been reported for non-substituted *N*-phenylformamide (Omondi *et al.*, 2008; Gowda *et al.*, 2009).

In the crystal, molecules are connected by intermolecular hydrogen bonding between the amide groups to form helical 2_1_ chains in the c axis direction (Table1, Fig. 2). The molecules are also connected by a weak intermolecular formamide-benzene C—H···π interaction. The C—H vector of this contact does not point to the midpoint of the acceptor ring, but points more closely to the C3—C4 bond. Similar hydrogen bonded chains occur in the solvent-free compound mentioned above. Molecules in adjacent chains are connected by a very weak intermolecular benzene-formamide C—H···O interaction to form a helix about a 6_1_ screw axis.

The crystal packing (Fig. 3) shows a channel along the c axis with an average radius of 3.71 Å and it is surrounded by isopropyl groups. Each channel contains two toluene solvate molecules per unit cell, as estimated by the SQUEEZE routine in *PLATON* (Spek, 2009).

### Experimental

*N*-(2,6-diisopropylphenyl)formamide was obtained as a byproduct from the synthesis of 1,3-bis-(2,6-diisopropylphenyl)imidazolium chloride (Hintermann, 2007). It can also be synthesized as reported by Krishnamurthy (1982). Crystallization from toluene resulted in the formation of colourless rod-shaped crystals of the title compound. To confirm the toluene contents of the sample, some single crystals were dissolved in CDCl_3_. ^1^H-NMR spectra of this solution showed the resonances of the major and minor rotamer of *N*-(2,6-diisopropylphenyl)formamide (Chitanda *et al.*, 2008) and also the resonances of toluene.

### Refinement

Friedel opposites were merged. An arbitrary direction of the polar axis was choosen. The crystal packing shows a channel about [0 0 1] with a volume of 366 Å^3^. Thus the channel has an effective diameter of 7.43 Å. Only diffuse electron density with a maximum of 0.68 e.Å^-3^ was found in the channel. The SQUEEZE routine in *PLATON* (Spek, 2009) was used to subtract the solvent contribution from the observed reflection intensities. The solvent electron count in the channel was calculated as 100 electrons/cell. Assuming the solvent to be toluene, there would be two grossly disordered toluene molecules in the unit cell. The NH H atom was located in a difference electron-density map and freely refined. The C-bound H atoms were included in calculated positions and treated as riding atoms: C-H = 0.95, 0.98 and 1.00Å for CH(aromatic), CH_3_ and CH(methine) H atoms, respectively, with U_iso_(H) = k × U_eq_(parent C-atom), where k = 1.5 for CH_3_ H-atoms and = 1.2 for other H-atoms.

### Crystal data

**Table:**

C_13_H_19_NO·0.33C_7_H_8_	*D*_x_ = 1.124 Mg m^−^^3^
*M**_r_* = 236.00	Mo *K*α radiation, λ = 0.71073 Å
Hexagonal, *P*6_1_	Cell parameters from 8192 reflections
Hall symbol: P 61	θ = 3–24°
*a* = 16.9133 (6) Å	µ = 0.07 mm^−^^1^
*c* = 8.4451 (4) Å	*T* = 185 K
*V* = 2092.2 (2) Å^3^	Rod, colourless
*Z* = 6	0.65 × 0.20 × 0.19 mm
*F*(000) = 772	

### Data collection

**Table:**

Siemens SMART 1K CCD diffractometer	1748 independent reflections
Radiation source: normal-focus sealed tube	1503 reflections with *I* > 2σ(*I*)
Graphite monochromator	*R*_int_ = 0.057
ω scans	θ_max_ = 28.0°, θ_min_ = 2.4°
Absorption correction: multi-scan (*SADABS*; Sheldrick, 2000)	*h* = −21→21
*T*_min_ = 0.933, *T*_max_ = 0.987	*k* = −22→21
23771 measured reflections	*l* = −11→10

### Refinement

**Table:**

Refinement on *F*^2^	Primary atom site location: structure-invariant direct methods
Least-squares matrix: full	Secondary atom site location: difference Fourier map
*R*[*F*^2^ > 2σ(*F*^2^)] = 0.058	Hydrogen site location: inferred from neighbouring sites
*wR*(*F*^2^) = 0.125	H atoms treated by a mixture of independent and constrained refinement
*S* = 1.08	*w* = 1/[σ^2^(*F*_o_^2^) + (0.06*P*)^2^ + 0.4*P*] where *P* = (*F*_o_^2^ + 2*F*_c_^2^)/3
1748 reflections	(Δ/σ)_max_ < 0.001
144 parameters	Δρ_max_ = 0.16 e Å^−^^3^
1 restraint	Δρ_min_ = −0.16 e Å^−^^3^

### Special details

**Table:**

Geometry. All e.s.d.'s (except the e.s.d. in the dihedral angle between two l.s. planes) are estimated using the full covariance matrix. The cell e.s.d.'s are taken into account individually in the estimation of e.s.d.'s in distances, angles and torsion angles; correlations between e.s.d.'s in cell parameters are only used when they are defined by crystal symmetry. An approximate (isotropic) treatment of cell e.s.d.'s is used for estimating e.s.d.'s involving l.s. planes.
Refinement. Refinement of *F*^2^ against ALL reflections. The weighted *R*-factor *wR* and goodness of fit *S* are based on *F*^2^, conventional *R*-factors *R* are based on *F*, with *F* set to zero for negative *F*^2^. The threshold expression of *F*^2^ > σ(*F*^2^) is used only for calculating *R*-factors(gt) *etc*. and is not relevant to the choice of reflections for refinement. *R*-factors based on *F*^2^ are statistically about twice as large as those based on *F*, and *R*- factors based on ALL data will be even larger.

### Fractional atomic coordinates and isotropic or equivalent isotropic displacement parameters (Å^2^)

**Table:**

	*x*	*y*	*z*	*U*_iso_*/*U*_eq_	
O1	0.08661 (11)	0.55915 (13)	0.1114 (2)	0.0375 (5)	
N1	−0.02094 (15)	0.50925 (15)	0.3050 (3)	0.0315 (5)	
H1A	−0.0291 (19)	0.496 (2)	0.397 (4)	0.034 (8)*	
C1	−0.09762 (16)	0.49482 (18)	0.2086 (3)	0.0297 (6)	
C2	−0.16677 (16)	0.40479 (18)	0.1800 (3)	0.0323 (6)	
C3	−0.24197 (17)	0.3921 (2)	0.0909 (3)	0.0410 (7)	
H3A	−0.2899	0.3320	0.0689	0.049*	
C4	−0.24742 (19)	0.4656 (2)	0.0349 (4)	0.0464 (8)	
H4A	−0.2995	0.4556	−0.0238	0.056*	
C5	−0.17845 (19)	0.5530 (2)	0.0628 (4)	0.0458 (7)	
H5A	−0.1830	0.6028	0.0212	0.055*	
C6	−0.10153 (17)	0.57032 (19)	0.1513 (3)	0.0375 (6)	
C7	−0.15810 (18)	0.32460 (18)	0.2384 (3)	0.0387 (6)	
H7A	−0.1271	0.3417	0.3438	0.046*	
C8	−0.2497 (2)	0.2370 (2)	0.2604 (4)	0.0532 (8)	
H8A	−0.2893	0.2495	0.3281	0.080*	
H8B	−0.2398	0.1904	0.3103	0.080*	
H8C	−0.2789	0.2150	0.1570	0.080*	
C9	−0.0972 (2)	0.3076 (2)	0.1255 (4)	0.0480 (8)	
H9A	−0.0378	0.3636	0.1155	0.072*	
H9B	−0.1263	0.2900	0.0212	0.072*	
H9C	−0.0884	0.2585	0.1676	0.072*	
C10	−0.02693 (19)	0.6683 (2)	0.1845 (4)	0.0443 (7)	
H10A	0.0267	0.6660	0.2289	0.053*	
C11	−0.0572 (3)	0.7136 (2)	0.3082 (6)	0.0721 (11)	
H11A	−0.0756	0.6768	0.4052	0.108*	
H11B	−0.1090	0.7181	0.2670	0.108*	
H11C	−0.0065	0.7748	0.3317	0.108*	
C12	0.0049 (3)	0.7257 (3)	0.0336 (6)	0.0755 (12)	
H12A	0.0159	0.6919	−0.0494	0.113*	
H12B	0.0615	0.7830	0.0551	0.113*	
H12C	−0.0423	0.7392	−0.0018	0.113*	
C13	0.06249 (17)	0.53788 (17)	0.2494 (3)	0.0319 (6)	
H13A	0.1072	0.5423	0.3224	0.038*	

### Atomic displacement parameters (Å^2^)

**Table:**

	*U*^11^	*U*^22^	*U*^33^	*U*^12^	*U*^13^	*U*^23^
O1	0.0290 (9)	0.0517 (11)	0.0258 (10)	0.0156 (9)	−0.0015 (8)	−0.0027 (8)
N1	0.0328 (12)	0.0378 (12)	0.0192 (11)	0.0141 (10)	−0.0025 (9)	−0.0013 (10)
C1	0.0236 (12)	0.0416 (14)	0.0241 (13)	0.0166 (10)	0.0014 (10)	−0.0033 (11)
C2	0.0269 (12)	0.0433 (14)	0.0218 (12)	0.0138 (11)	0.0042 (10)	−0.0044 (11)
C3	0.0291 (13)	0.0561 (17)	0.0313 (15)	0.0163 (13)	−0.0002 (12)	−0.0094 (14)
C4	0.0287 (14)	0.075 (2)	0.0391 (16)	0.0288 (15)	−0.0051 (12)	−0.0067 (15)
C5	0.0412 (16)	0.0625 (18)	0.0452 (18)	0.0345 (15)	0.0027 (14)	0.0055 (15)
C6	0.0311 (13)	0.0508 (16)	0.0337 (15)	0.0227 (12)	0.0050 (11)	0.0009 (13)
C7	0.0377 (14)	0.0409 (15)	0.0301 (14)	0.0141 (12)	−0.0028 (12)	−0.0046 (12)
C8	0.0514 (18)	0.0426 (16)	0.0497 (18)	0.0117 (14)	0.0077 (16)	−0.0040 (15)
C9	0.0461 (16)	0.0455 (16)	0.0511 (19)	0.0219 (13)	0.0010 (15)	−0.0035 (15)
C10	0.0391 (15)	0.0446 (16)	0.0546 (19)	0.0250 (13)	0.0054 (14)	0.0076 (15)
C11	0.064 (2)	0.0493 (18)	0.082 (3)	0.0127 (16)	0.012 (2)	−0.016 (2)
C12	0.073 (2)	0.066 (2)	0.077 (3)	0.026 (2)	0.022 (2)	0.023 (2)
C13	0.0275 (12)	0.0368 (14)	0.0280 (13)	0.0134 (11)	−0.0070 (10)	−0.0054 (11)

### Geometric parameters (Å, º)

**Table:**

O1—C13	1.227 (3)	C7—H7A	1.0000
N1—C13	1.328 (4)	C8—H8A	0.9800
N1—C1	1.445 (3)	C8—H8B	0.9800
N1—H1A	0.80 (4)	C8—H8C	0.9800
C1—C6	1.398 (4)	C9—H9A	0.9800
C1—C2	1.401 (4)	C9—H9B	0.9800
C2—C3	1.399 (4)	C9—H9C	0.9800
C2—C7	1.517 (4)	C10—C11	1.527 (5)
C3—C4	1.375 (4)	C10—C12	1.528 (5)
C3—H3A	0.9500	C10—H10A	1.0000
C4—C5	1.371 (4)	C11—H11A	0.9800
C4—H4A	0.9500	C11—H11B	0.9800
C5—C6	1.399 (4)	C11—H11C	0.9800
C5—H5A	0.9500	C12—H12A	0.9800
C6—C10	1.525 (4)	C12—H12B	0.9800
C7—C8	1.528 (4)	C12—H12C	0.9800
C7—C9	1.532 (4)	C13—H13A	0.9500
			
C13—N1—C1	124.2 (2)	C7—C8—H8C	109.5
C13—N1—H1A	117 (2)	H8A—C8—H8C	109.5
C1—N1—H1A	119 (2)	H8B—C8—H8C	109.5
C6—C1—C2	122.6 (2)	C7—C9—H9A	109.5
C6—C1—N1	119.3 (2)	C7—C9—H9B	109.5
C2—C1—N1	118.1 (2)	H9A—C9—H9B	109.5
C3—C2—C1	117.4 (2)	C7—C9—H9C	109.5
C3—C2—C7	121.6 (2)	H9A—C9—H9C	109.5
C1—C2—C7	120.9 (2)	H9B—C9—H9C	109.5
C4—C3—C2	120.9 (3)	C6—C10—C11	111.6 (2)
C4—C3—H3A	119.6	C6—C10—C12	112.0 (3)
C2—C3—H3A	119.6	C11—C10—C12	110.6 (3)
C5—C4—C3	120.7 (3)	C6—C10—H10A	107.5
C5—C4—H4A	119.7	C11—C10—H10A	107.5
C3—C4—H4A	119.7	C12—C10—H10A	107.5
C4—C5—C6	121.2 (3)	C10—C11—H11A	109.5
C4—C5—H5A	119.4	C10—C11—H11B	109.5
C6—C5—H5A	119.4	H11A—C11—H11B	109.5
C1—C6—C5	117.2 (3)	C10—C11—H11C	109.5
C1—C6—C10	122.5 (2)	H11A—C11—H11C	109.5
C5—C6—C10	120.2 (3)	H11B—C11—H11C	109.5
C2—C7—C8	113.7 (2)	C10—C12—H12A	109.5
C2—C7—C9	109.9 (2)	C10—C12—H12B	109.5
C8—C7—C9	110.2 (2)	H12A—C12—H12B	109.5
C2—C7—H7A	107.6	C10—C12—H12C	109.5
C8—C7—H7A	107.6	H12A—C12—H12C	109.5
C9—C7—H7A	107.6	H12B—C12—H12C	109.5
C7—C8—H8A	109.5	O1—C13—N1	125.4 (2)
C7—C8—H8B	109.5	O1—C13—H13A	117.3
H8A—C8—H8B	109.5	N1—C13—H13A	117.3
			
C13—N1—C1—C6	−73.6 (3)	N1—C1—C6—C10	0.9 (4)
C13—N1—C1—C2	108.2 (3)	C4—C5—C6—C1	0.6 (4)
C6—C1—C2—C3	−0.3 (4)	C4—C5—C6—C10	−178.3 (3)
N1—C1—C2—C3	177.9 (2)	C3—C2—C7—C8	−26.6 (4)
C6—C1—C2—C7	177.4 (2)	C1—C2—C7—C8	155.8 (2)
N1—C1—C2—C7	−4.4 (3)	C3—C2—C7—C9	97.4 (3)
C1—C2—C3—C4	−0.3 (4)	C1—C2—C7—C9	−80.1 (3)
C7—C2—C3—C4	−178.0 (3)	C1—C6—C10—C11	−105.1 (3)
C2—C3—C4—C5	1.1 (4)	C5—C6—C10—C11	73.8 (4)
C3—C4—C5—C6	−1.2 (5)	C1—C6—C10—C12	130.3 (3)
C2—C1—C6—C5	0.2 (4)	C5—C6—C10—C12	−50.8 (4)
N1—C1—C6—C5	−178.0 (2)	C1—N1—C13—O1	3.0 (4)
C2—C1—C6—C10	179.1 (3)		

### Hydrogen-bond geometry (Å, º)

**Table:**

*D*—H···*A*	*D*—H	H···*A*	*D*···*A*	*D*—H···*A*
N1—H1*A*···O1^i^	0.80 (4)	2.05 (4)	2.826 (3)	164 (3)
C4—H4*A*···O1^ii^	0.95	2.56	3.418 (3)	150
C13—H13*A*···C3^i^	0.95	3.01	3.917 (4)	161
C13—H13*A*···C4^i^	0.95	3.03	3.973 (4)	173

Symmetry codes: (i) −*x*, −*y*+1, *z*+1/2; (ii) *y*−1, −*x*+*y*, *z*−1/6.
